# Insights into the effect of exposed Mn_2_O_3_ crystal planes on toluene oxidation over MnO_*x*_/IM monolithic catalysts

**DOI:** 10.1039/d6ra07826e

**Published:** 2026-11-02

**Authors:** Kewang Huang, Jianshen Wang, Gangguo Zhao, Xiaojing Gui, Dan Liu, Jianzhou Gui

**Affiliations:** a State Key Laboratory of Separation Membranes and Membrane Processes, Tianjin Key Laboratory of Green Chemical Technology and Process Engineering, School of Material Science and Engineering, Tiangong University Tianjin 300387 China liudan@tiangong.edu.cn guijianzhou@tiangong.edu.cn; b Ningxia Coal Industry Co. Ltd, CHN ENERGY Yinchuan 750011 P. R. China; c Shandong Haihua Co., Ltd Weifang Shandong 262737 China; d School of Chemical Engineering and Technology, Tiangong University Tianjin 300387 China

## Abstract

*In situ* growth of Mn_2_O_3_ on the surface of the iron mesh was performed hydrothermally to prepare novel catalysts with enhanced toluene oxidation performance. The effect of exposed crystal planes of catalysts, regulated by manganese concentration, on the catalytic performance of toluene oxidation was systematically studied using various characterization techniques, including XRD, SEM, TEM, Raman, H_2_-TPR, and XPS. The 1.2MnO_*x*_/IM catalyst exhibited superior toluene oxidation performance, attributed to Mn_2_O_3_ (111)- facet-induced synergistic effects between its super-low-temperature oxidation capability and abundant oxygen vacancies. DFT calculations indicated that the catalyst with an exposed Mn_2_O_3_ (111) crystal plane has stronger toluene/O_2_ adsorption and weaker CO_2_ adsorption, demonstrating its excellent catalytic activity and stability. The work opens an exploration of the role of the crystal plane in monolithic catalysts and provides a theoretical basis for monolithic catalysts for VOC oxidation.

## Abstract 2

Adjusting Mn(NO_3_)_2_ concentration enhances Mn_2_O_3_ (111) facet exposure on iron mesh and improves toluene oxidation performance.

## Graphical abstract

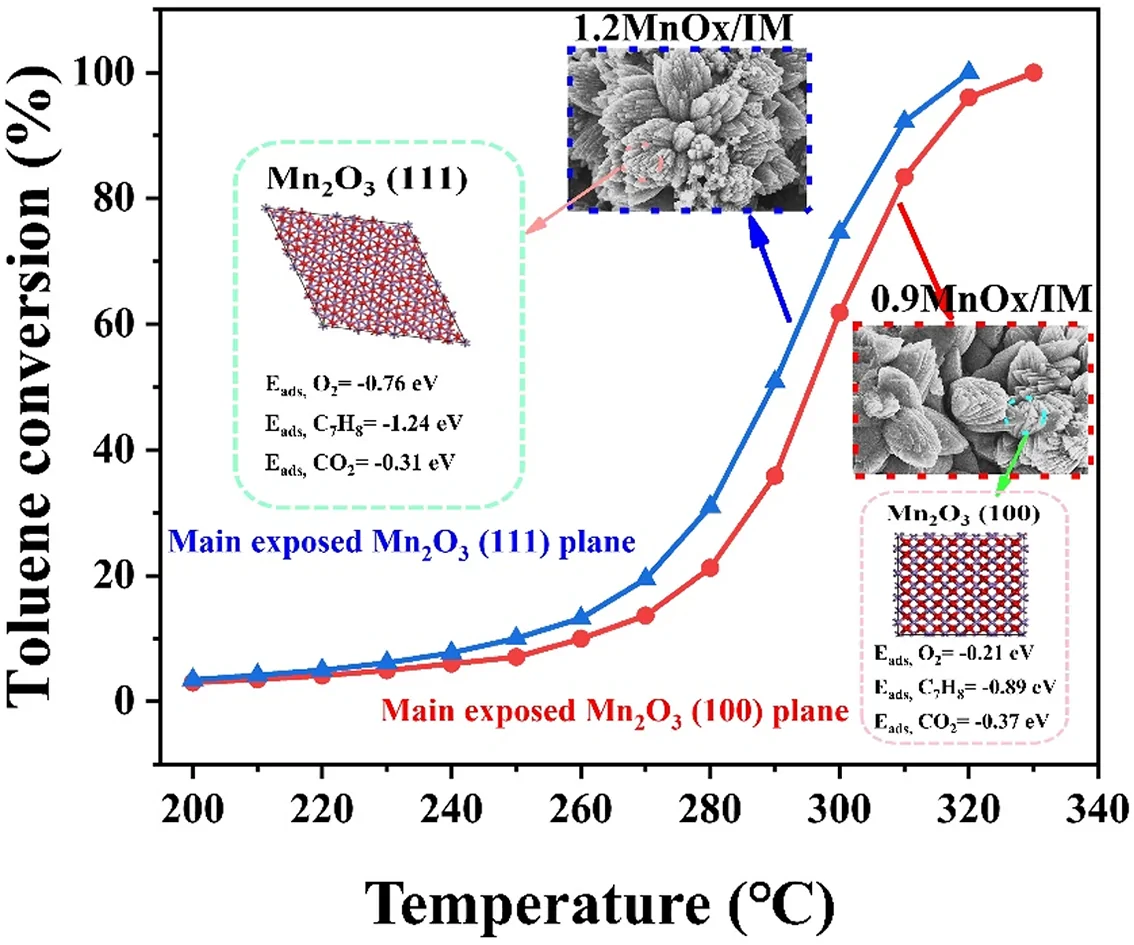

## 1. Introduction

Volatile organic compounds (VOCs), pollutants that contaminate the environment and harm people's health,^1–3^ mainly include alkanes,^4–6^ olefins,^1^ heteroatom-containing VOCs (such as O, N, Cl),^7–9^ and aromatic VOCs (such as benzene, toluene, xylene).^10–12^ Thus, it is crucial to remove them efficiently. Compared with absorption, adsorption, membrane separation, and plasma elimination,^13–15^ catalytic oxidation is an efficient method, and its key lies in designing catalysts with higher catalytic performance. Metal oxide-based catalysts have attracted significant research attention, and activating lattice oxygen to enhance catalytic activity remains a scientific challenge.^16,17^

As for manganese oxides, with their advantages of polymorphism and polyvalence, researchers are particularly interested in enhancing their catalytic activity for VOC oxidation by doping with other metals or by regulating their crystal phase, crystal plane, and morphology.^18–20^ Wu *et al.* indicated that doping Zr into the MnO_2_ lattice generated excellent redox activity and more oxygen vacancies, thereby enhancing the catalytic activity.^21^ An O-vacancy-rich porous MnO_2_ nanosheet catalyst provided more active oxygen species to enhance oxygen mobility and reactivity, thus improving the catalytic activity of propane oxidation.^22^ In Wang *et al.*'s research, nanorod-like α-MnO_2_ had excellent catalytic performance.^23^ Even for the same phase of MnO_2_, crystallinity and morphology also affect the catalytic activity for toluene oxidation. Moreover, different exposed crystal planes in MnO_*x*_ catalysts have different oxidation abilities.^24^ The work of Huang *et al.* suggested that the three-dimensional (3D) hierarchical cube-like morphology, mainly exposed to the (211) crystal plane, exhibited the best catalytic activity for benzene oxidation.^25^ Bi *et al.* indicated that the catalyst with the main exposed MnO_2_ (110) plane performed the best catalytic activity and stability in propane oxidation.^26^ The research results of Chi *et al.* indicated that the α-MnO_2_ catalyst with an exposed (310) facet exhibited the highest catalytic activity and excellent stability during soot oxidation.^27^ Based on the above description, it can be concluded that regulating the exposed crystal plane of Mn_2_O_3_ catalysts is a promising approach to enhance catalytic performance.

Monolithic catalysts, in which the active component is supported by a monolithic honeycombed cordierite,^28^ metal mesh,^29^ or metal foam,^30^ have the advantage of low pressure drop, uniform heat distribution, and conducive mass and heat transfer.^31,32^ To our knowledge, there is little literature on the preparation of different exposed crystal planes and their effects on the catalytic performance of VOC oxidation. Thus, it is necessary to prepare MnO_*x*_ monolithic catalysts with different exposed MnO_*x*_ crystal planes and to study this important scientific issue.

To address the problem proposed above, this work reports a series of MnO_*x*_ catalysts with different exposed crystal planes, grown on the surface of iron mesh *via* the hydrothermal method. Toluene, a common VOC pollutant, was selected for studying VOC oxidation. The effect of exposed crystal planes on the catalytic performance of toluene oxidation was systematically studied by a series of characterizations, including XRD, SEM, TEM, Raman, H_2_-TPR, and XPS. The adsorption energies (*E*_ads_) of toluene, O_2,_ and CO_2_ were also calculated to gain insight into the better catalytic activity. While Mn loading, morphology, and pore structure concurrently influence activity, we demonstrate that the (111) facet serves as the dominant geometric factor that intrinsically promotes oxygen vacancy formation and low-temperature reducibility, thereby governing the overall catalytic behavior. The work opens an exploration of the role of the crystal plane in monolithic catalysts and provides a theoretical basis for monolithic catalysts for VOC oxidation.

## 2. Experimental section

### 2.1 Chemical reagents

All reagents are analytically pure. The Q304-type iron mesh was purchased from Shanghai Fine Filter Equipment Co., Ltd. Manganese nitrate tetrahydrate, urea, and ammonium fluoride were obtained from Tianjin Kermiou Chemical Reagent Co., Ltd.

### 2.2 Synthesis of catalysts

Catalysts were synthesized according to previously reported methods with minor modifications.^33^ For the preparation of iron mesh (IM)-based monolithic catalysts, an iron wire mesh of Q304 type was used. First, the mesh was cut into rectangular pieces of 3.5 × 1.5 cm. The iron mesh pieces were then ultrasonically cleaned with deionized water and ethanol, followed by 10 min of acid pickling to remove the surface rust and mill scale. A certain concentration of manganese nitrate tetrahydrate (0.9 mmol, 1.2 mmol, 1.5 mmol), urea (24 mmol), and ammonium fluoride (4.8 mmol) was dissolved in 40 mL of deionized water to form a homogeneous solution. Two pieces of acid-pickled iron mesh were immersed in the hydrolysis solution, transferred to a Teflon-lined stainless-steel autoclave, and maintained at 80 °C for 12 h. The obtained samples were rinsed several times with deionized water and ethanol after cooling to room temperature. After drying at 80 °C, the samples were calcined at 400 °C for 5 h with a heating rate of 2 °C min^−1^ in a muffle furnace. The synthesized samples were denoted as 0.9MnO_*x*_/IM, 1.2MnO_*x*_/IM, and 1.5MnO_*x*_/IM, respectively. The sample obtained by the same steps, except without adding a manganese source, was denoted as IM.

### 2.3 Activity test

The catalytic performance of catalysts for toluene oxidation was tested in a fixed-bed quartz tube (i.d. = 4 mm, length = 450 mm). A piece of catalyst (0.3 g, about 0.03 g Mn), rolled into a cylindrical shape with dense contact, was put in the middle of a quartz tube and fixed with quartz wool. A flow (20 vol% O_2_/N_2_, 90 mL min^−1^) containing 1000 ppm of toluene was passed through the reactor from top to bottom, where toluene vapor was controlled by blowing dry air into a liquid toluene saturator. Reaction temperatures were measured at the bottom of the bed layer. The toluene conversion and CO_2_ yield were measured by *in situ* mass spectrometry. Subsequently, the long-term stability of the 1.2MnO_*x*_/IM catalyst was tested for 10 hours at 310 °C.

The calculation methods for toluene conversion and CO_2_ yield were listed in SI S1.

### 2.4 Characterization

The characterizations of ICP-OES, XRD, SEM, HRTEM, Raman, XPS, H_2_-TPR, O_2_-TPD, *in situ* DRIFTS, and DFT calculations were described in SI S2.

## 3. Results and discussion

### 3.1 Structural properties of catalysts

According to the results of XRD patterns in Fig. 1, all MnO_*x*_/IM catalysts show the crystal phase of Mn_2_O_3_ (JCPDS 41-1442).^34^ Compared with the pure IM sample, the peak intensities of IM in all MnO_*x*_/IM catalysts become weaker, meaning MnO_*x*_ grows on the surface of IM. After calculation, the peak intensity ratios of (111), (100), and (211) surfaces in all MnO_*x*_/IM catalysts are 1 : 0.5 : 0.4 in 0.9MnO_*x*_/IM catalyst, 1 : 0.3 : 0.3 in 1.2MnO_*x*_/IM catalyst, and 1 : 0.4 : 0.5 in 1.5MnO_*x*_/IM catalyst, meaning three MnO_*x*_/IM catalysts mainly expose Mn_2_O_3_ (111) plane, Mn_2_O_3_ (100) plane, and Mn_2_O_3_ (211) plane, respectively.

**Fig. 1:**
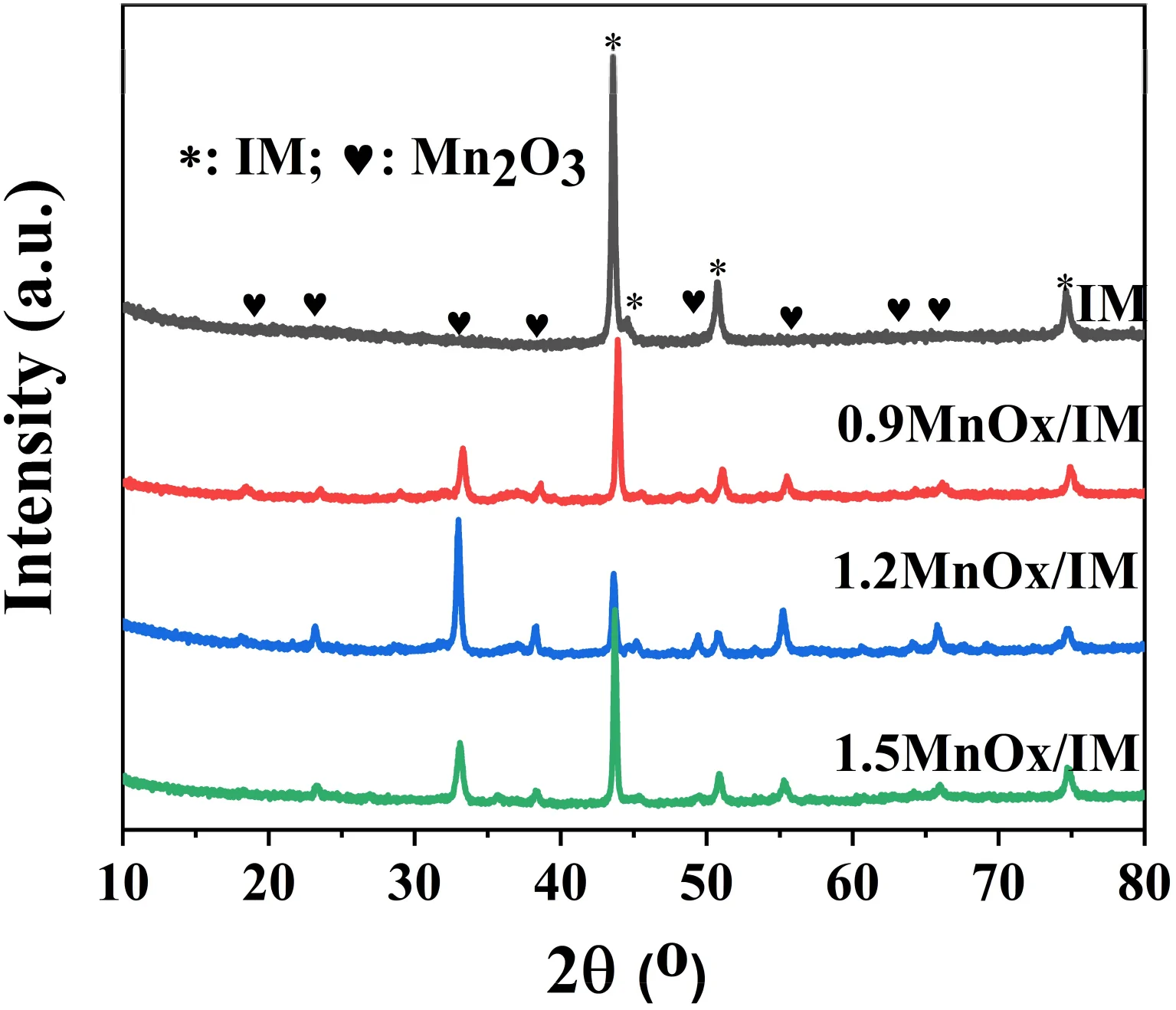
XRD patterns of all samples.

In SEM images of Fig. 2A–D, the pure IM surface is rough, while all MnO_*x*_/IM catalysts show a tightly packed structure with vertically arranged sheets, accompanied by differences in sheet thickness, meaning the exposure of different crystal planes. The exposure of different crystal planes in MnO_*x*_/IM catalysts was further proved by HRTEM images in Fig. 2E–G. The lattice fringe spacings of 0.47 nm, 0.24 nm, and 0.20 nm are attributed to the Mn_2_O_3_ (100) crystal plane, Mn_2_O_3_ (111) crystal plane, and Mn_2_O_3_ (211) crystal plane, respectively, which indicate the exposure of different crystal planes in MnO_*x*_/IM catalysts. The blurry lattice fringe in 1.2MnO_*x*_/IM may be related to oxygen vacancies. The results of the N_2_ adsorption–desorption isotherm are shown in Fig. S1 and Table S1. A relatively larger average pore diameter in the 1.2MnO_*x*_/IM catalyst with an exposed Mn_2_O_3_ (111) plane can be observed, which is beneficial for the diffusion of reactant molecules. The larger pore diameter and nanosheet morphology of 1.2MnO_*x*_/IM are facet-derived consequences of anisotropic growth directed by the (111) plane.

**Fig. 2:**
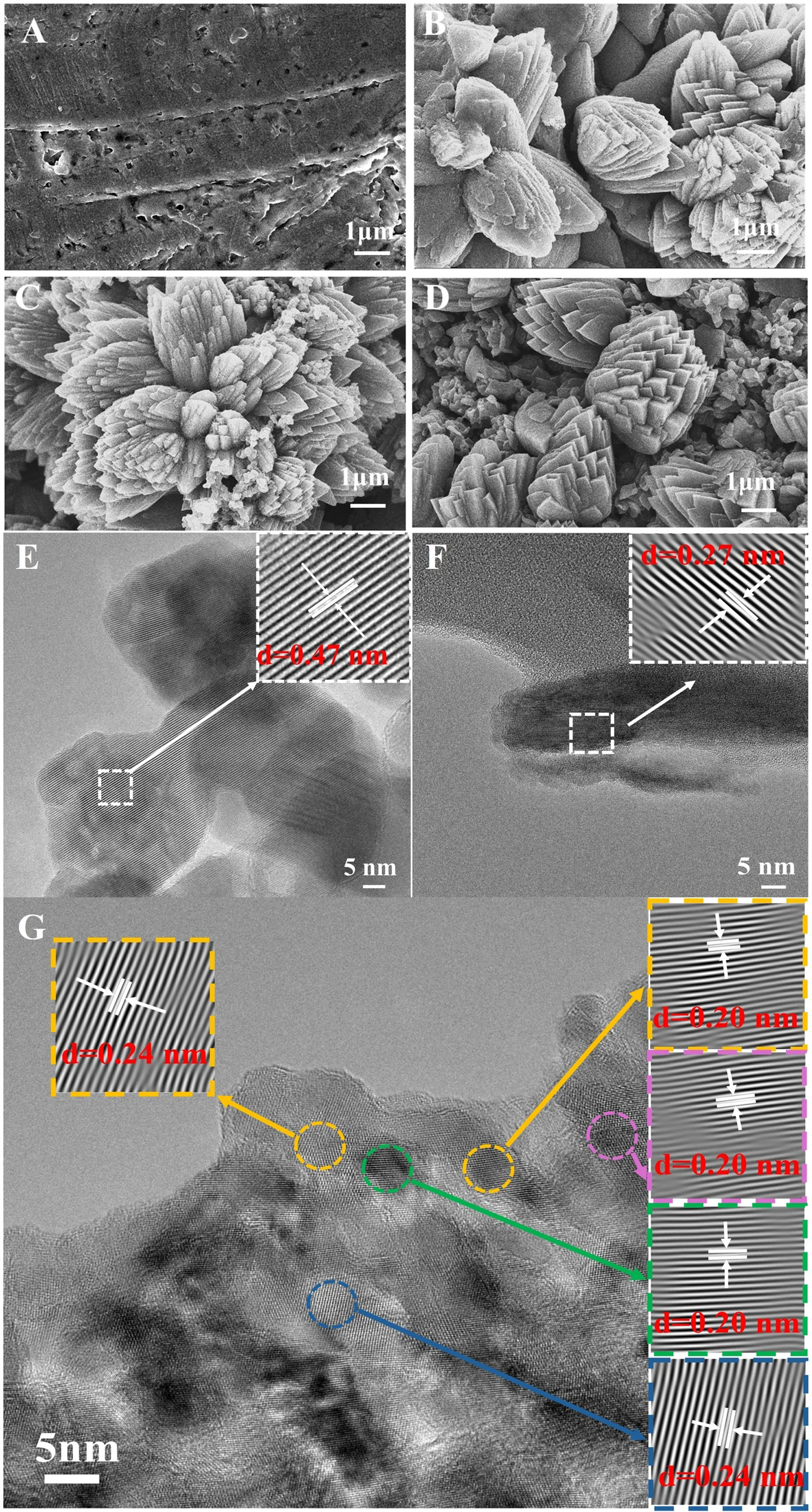
SEM images of IM (A), 0.9MnO_*x*_/IM (B), 1.2MnO_*x*_/IM (C), and 1.5MnO_*x*_/IM (D); HRTEM images of 0.9MnO_*x*_/IM (E), 1.2MnO_*x*_/IM (F), 1.5MnO_*x*_/IM (G).

As shown in Fig. S2A–F, the additional HRTEM images reveal that the 0.27 nm lattice fringes assigned to Mn_2_O_3_ (111) are more frequently observed for 1.2MnO_*x*_/IM, suggesting a higher exposure of the (111) facet.

The peak between 635 cm^−1^ and 655 cm^−1^ in Raman spectra (Fig. 3) is attributed to the Mn^3+^-O symmetric stretching vibrations in the [MnO_6_] octahedral units.^35^ Compared with 0.9MnO_*x*_/IM and 1.5MnO_*x*_/IM catalysts, the peak in the 1.2MnO_*x*_/IM catalyst shifts to a lower wavenumber, meaning the presence of coordinatively unsaturated Mn^3+^ species, which is related to more oxygen vacancies in the 1.2MnO_*x*_/IM catalyst.

**Fig. 3:**
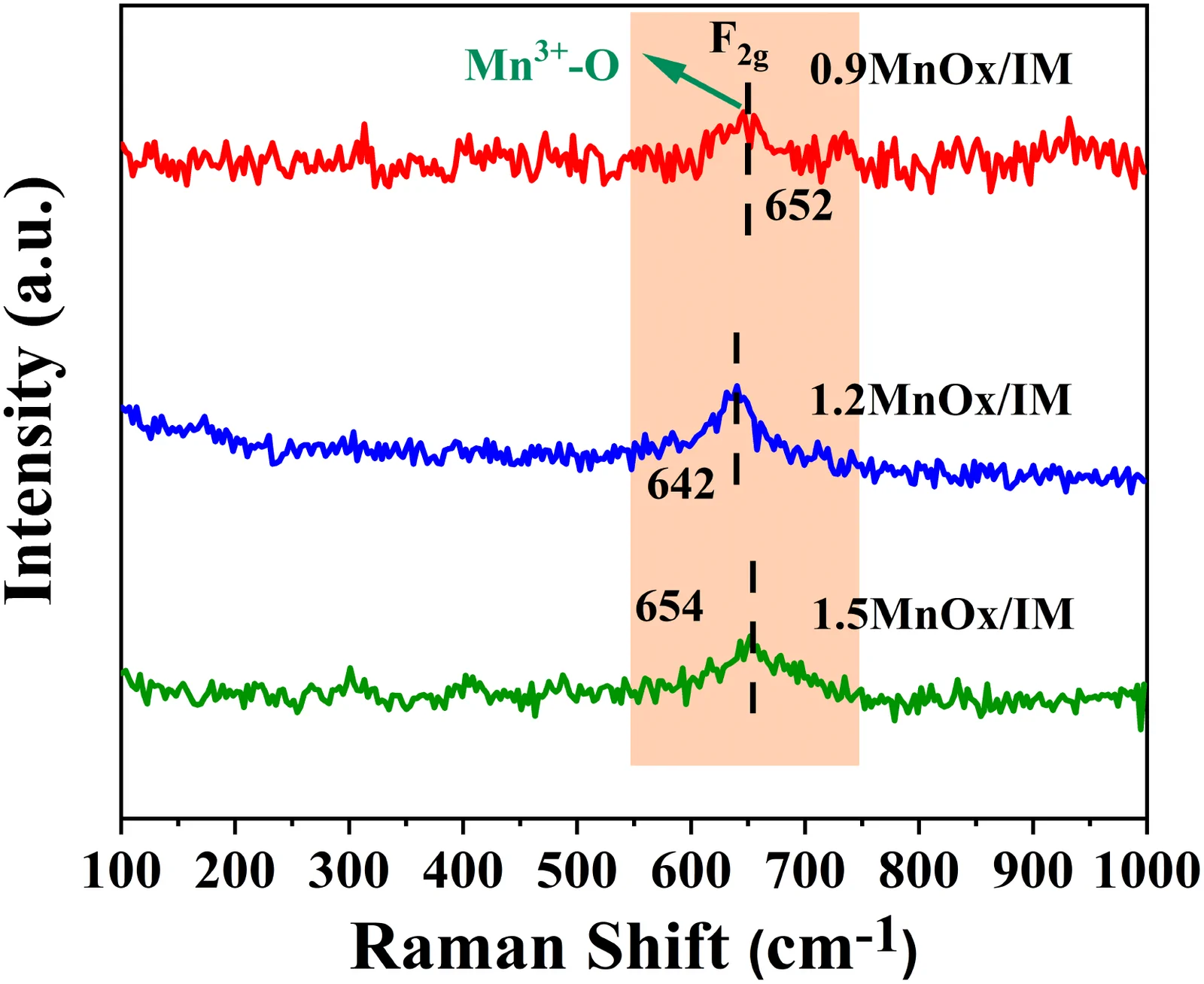
Raman spectra of all MnO_*x*_/IM catalyst samples.

The surface chemical states of manganese and oxygen on the catalyst surface have been studied by XPS analysis, as shown in Fig. 4. The peaks at 641.2 eV and 653.1 eV in Fig. 4A are attributed to Mn 2p_3/2_ and Mn 2p_1/2_, respectively. The peaks at 640.8 eV and 641.8 eV in the Mn 2p_3/2_ region are associated with Mn^2+^ and Mn^3+^ species.^18,36^ The peaks at 529.4 eV, 531.3 eV, 532.3 eV, and 533.0 eV in the O 1s spectrum (Fig. 4B) are ascribed to lattice oxygen species of iron oxides (O_Fe–O_), lattice oxygen species of manganese oxides (O_Mn–O_), oxygen vacancies (O_v_), and adsorbed water molecules (O_w_), respectively.^36–38^ The Mn^3+^/Mn^2+^ ratio and O_v_/O ratio in all MnO_*x*_/IM catalysts are shown in Table 1, indicating that the 1.2MnO_*x*_/IM catalyst has the highest concentration of Mn^3+^ species and the highest proportion of oxygen vacancies.

**Fig. 4:**
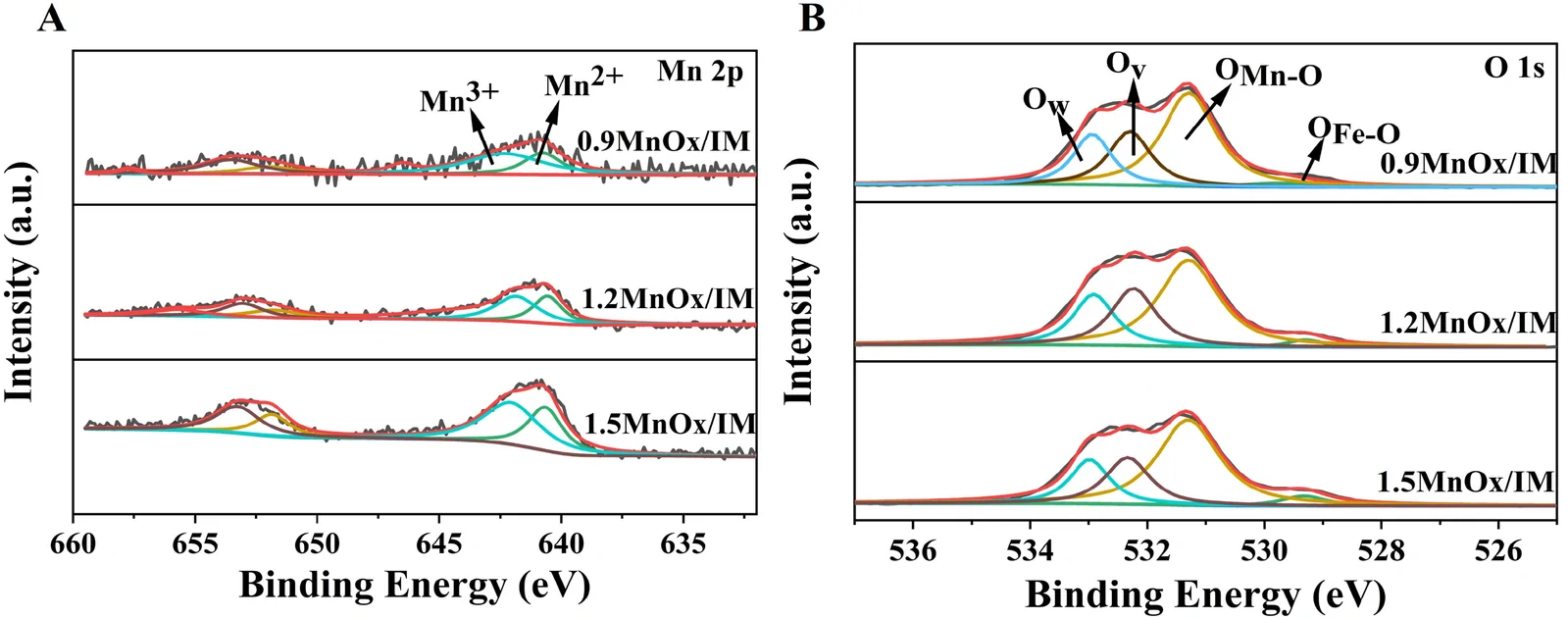
Mn 2p (A) and O 1s (B) XPS spectra.

**Table 1:** Mn content, surface chemical states, and TOF values of the MnO_*x*_/IM catalysts

Catalyst	Mn content^a^ (wt%)	Ov/O^b^	Mn^3+^/Mn^2+^	TOF_Mn_^c^ (×10^−3^ s ^−1^)
0.9MnO_*x*_/IM	1.9	0.24	0.67	3.21
1.2MnO_*x*_/IM	2.6	0.25	1.10	3.43
1.5MnO_*x*_/IM	3.2	0.22	0.61	1.01

a Mn content was calculated by ICP.

b O represents the total area of all oxygen species in O 1s XPS spectra of a certain catalyst.

c TOF was calculated based on the total Mn content, assuming that all Mn atoms were accessible to reactants at 285 °C.

### 3.2 Redox ability of catalysts

To indicate the reducibility of the MnO_*x*_/IM catalysts, H_2_-TPR results are shown in Fig. 5A. The reduction peaks between 300 °C and 400 °C in 0.9MnO_*x*_/IM and 1.2MnO_*x*_/IM are ascribed to the reduction of Mn_2_O_3_ to Mn_3_O_4_. The reduction peaks between 400 °C and 500 °C in 0.9MnO_*x*_/IM and 1.2MnO_*x*_/IM are ascribed to the reduction of Mn_3_O_4_ to MnO.^39–41^ While the reduction peaks from 300 °C to 500 °C and from 500 °C to 650 °C are associated with the catalytic reduction of Mn_2_O_3_ to Mn_3_O_4_ and Mn_3_O_4_ to MnO in 1.5MnO_*x*_/IM, respectively.^18,42^ Compared with 0.9MnO_*x*_/IM and 1.5MnO_*x*_/IM, the reduction peak in the 1.2MnO_*x*_/IM catalyst can be observed at a lower temperature, demonstrating that the 1.2MnO_*x*_/IM sample exhibits excellent reducibility, which is related to the presence of more oxygen vacancies in the 1.2MnO_*x*_/IM catalyst.

**Fig. 5:**
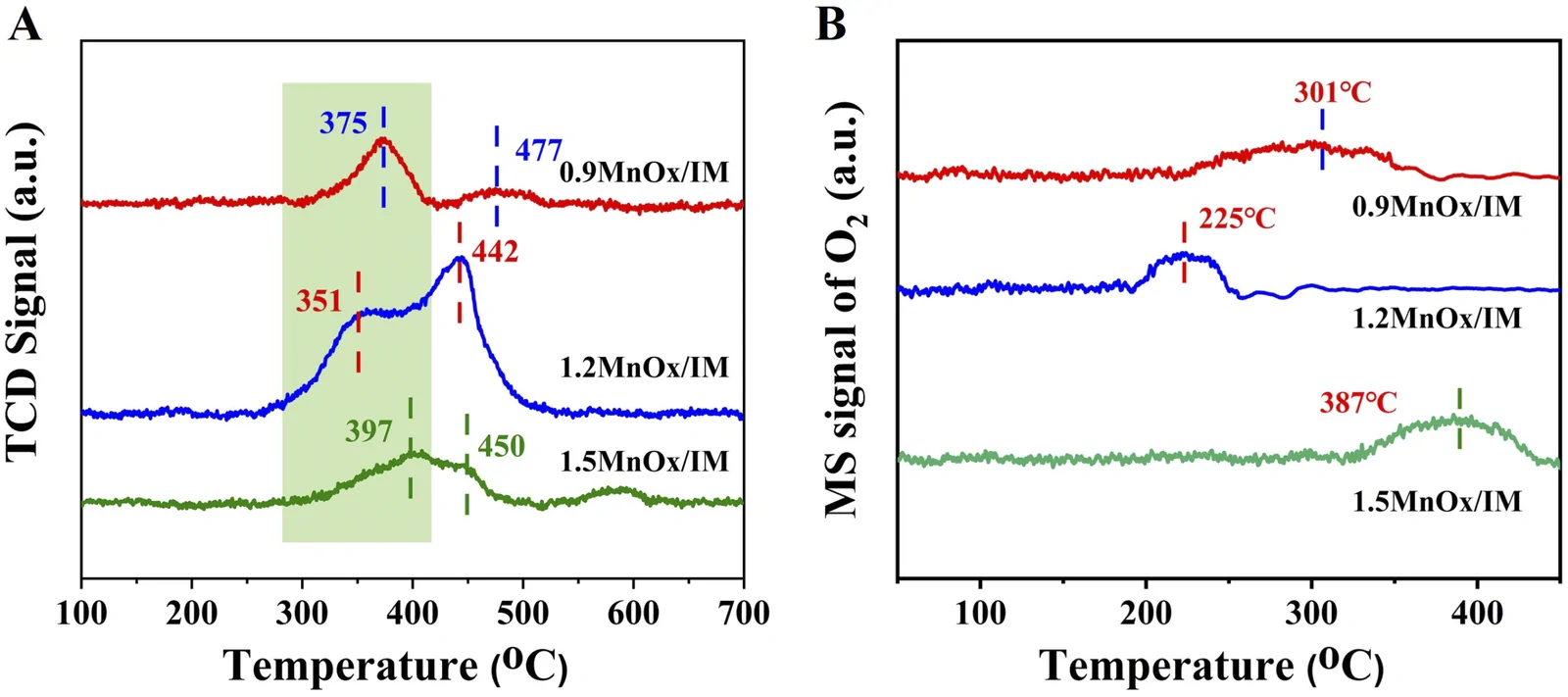
H_2_-TPR (A) and O_2_-TPD (B) images of all MnO_*x*_ catalysts.

O_2_-TPD curve probes the mobility of surface oxygen species (Fig. 5B). Each catalyst shows a single desorption peak at 229 °C for 1.2MnO_*x*_/IM, 290 °C for 0.9MnO_*x*_/IM, and 385 °C for 1.5MnO_*x*_/IM. Because all three desorption peaks appear below 400 °C, the desorbed oxygen is weakly bound surface oxygen at oxygen vacancies rather than bulk lattice oxygen. The large decrease in desorption peak temperature (for 1.2MnO_*x*_/IM, 61 °C lower than 0.9MnO_*x*_/IM, and 156 °C lower than 1.5MnO_*x*_/IM) suggests that 1.2MnO_*x*_/IM has the most mobile surface oxygen, consistent with its XPS results on higher oxygen-vacancy concentration.^43^

### 3.3 Catalytic performance

The conversion rate of toluene oxidation across all catalysts was shown in Fig. 6A, indicating that the 1.2MnO_*x*_/IM catalyst with exposed Mn_2_O_3_ (111) crystal planes shows the best catalytic activity. CO_2_ yield in Fig. 6B suggests that the 1.2MnO_*x*_/IM catalyst can completely oxidize toluene to CO_2_ at lower temperatures. Pure IM showed negligible catalytic activity (*T*_90_ > 400 °C, much higher than that of the MnO_*x*_/IM catalysts). This indicates that the contribution of the iron mesh substrate to toluene oxidation is negligible, and the catalytic activity mainly originates from the Mn_2_O_3_ phase. To further evaluate the catalytic activity, the toluene reaction rates were measured in the 270–290 °C range (Fig. 6C). The 1.2MnO_*x*_/IM catalyst showed the highest reaction rate, consistent with its best toluene conversion, further confirming its superior activity. To obtain insights into the intrinsic activity, the activation energy (*E*_a_) was calculated from Arrhenius plots at toluene conversion <20% for 0.9MnO_*x*_/IM, 1.2MnO_*x*_/IM, and 1.5MnO_*x*_/IM (Fig. 6D). Among these catalysts, 1.2MnO_*x*_/IM had the lowest *E*_a_, whereas 1.5MnO_*x*_/IM had the highest *E*_a_, corresponding to its poorest catalytic activity. In the previous literature researching typical iron mesh-based monolithic catalysts, the *T*_90_ of 0.1MnCF-LDO/IM (60 000 mL g^−1^ h^−1^)^44^ and 50CoFe-LDO/IM (10 000 h^−1^)^33^ was 316 °C and 348 °C, meaning the 1.2MnO_*x*_/IM catalyst with exposed Mn_2_O_3_ (111) crystal plane in our work has good catalytic performance at a space velocity of 180 000 mL g^−1^ h^−1^.

**Fig. 6:**
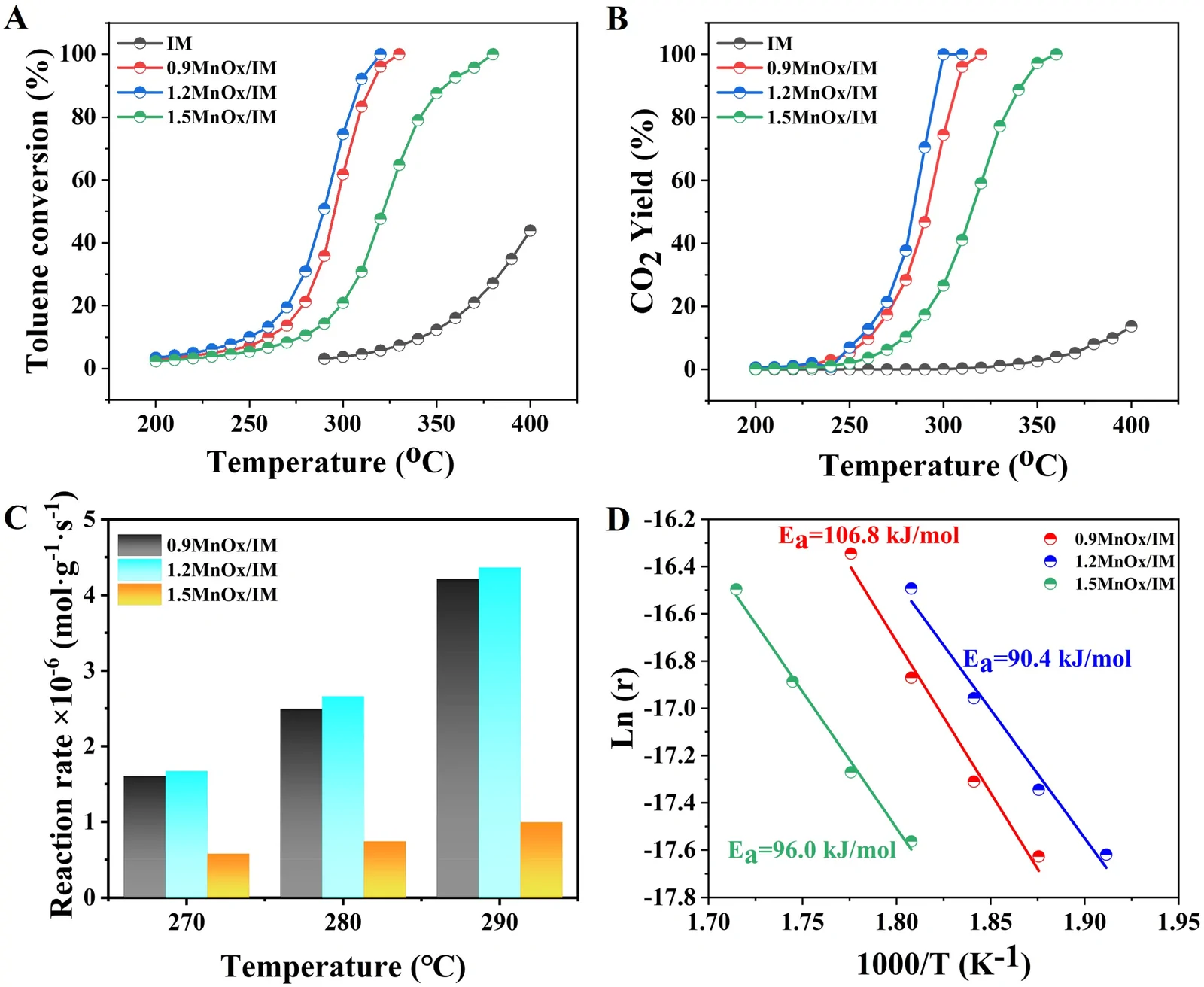
Catalytic performance (A), CO_2_ yield (B), Arrhenius plots (C), and the toluene reaction rate (D) of all samples.

To determine the supported Mn content in all MnO_*x*_/IM catalysts, ICP analysis was performed, and the results are reported in Table 1, indicating lower Mn content across all catalysts and excellent catalytic performance in toluene oxidation. Notably, the TOF values, which are also summarized in Table 1 and calculated based on the Mn content, demonstrate that catalytic performance is independent of Mn loading and is predominantly governed by the exposed Mn_2_O_3_ (111) crystal plane.

According to the results of H_2_-TPR, XPS, and Raman, the 1.2MnO_*x*_/IM catalyst rich in the exposed Mn_2_O_3_ (111) plane exhibits excellent low-temperature reducibility, more Mn^3+^ species, and oxygen vacancies, thus enhancing the catalytic activity and stability of toluene oxidation. According to Xin's research, the catalyst with the Mn_2_O_3_ (111) crystal plane exhibits higher oxidation activity at high temperatures,^35^ which is consistent with the results of this work.

To indicate the stability of the superior 1.2MnO_*x*_/IM catalyst, a long-term toluene oxidation experiment on the catalyst was carried out at 310 °C for 50 hours, and the results were shown in Fig. 8A. The catalytic performance can be consistently maintained at 90%, suggesting the 1.2MnO_*x*_/IM catalyst has outstanding stability. To evaluate the stability of the catalyst used, XRD, SEM, and XPS analyses were performed. Its XRD pattern (Fig. 8B) is nearly identical to that of the fresh catalyst, with unchanged peak positions and relative intensities and no peaks from impurity phases or phase transformation, indicating a well-preserved bulk structure. SEM images (Fig. 8C) show that the original particle morphology and size distribution are retained, with no evidence of aggregation, sintering, or framework collapse. XPS further shows largely preserved surface states: the Mn^3+^/Mn^2+^ ratio decreases only slightly from 1.10 to 1.09 (Mn 2p, Fig. S3A), and the O_v_/O ratio decreases from 0.25 to 0.23 (O 1s, Fig. S3B), both of which were listed in Table S2. Overall, the catalyst maintains good bulk and surface stability under the reaction conditions.

### 3.4 Catalytic mechanism

To trace the reaction pathway and identify the surface intermediates formed over the MnO_*x*_ catalysts, *in situ* DRIFTS spectra were collected at 310 °C as a function of reaction time (Fig. 7A–C). The bands at 1491, 1525, and 1599 cm^−1^, which arise from aromatic-ring vibrations, indicate that toluene or a phenyl-containing species derived from it is adsorbed on the catalyst surface.^45^ The bands at 1025 and 1174 cm^−1^, assigned to the C–O stretching of alkoxide species, show that adsorbed toluene is converted into benzyl alcohol through scission of the C–H bond of the methyl group.^46^ The bands at 1417, 1538, and 1550 cm^−1^, which originate from the carboxylate group, the former from its symmetric and the latter two from its asymmetric –COO– stretching vibrations, demonstrate the formation of benzoates, whereas the band at 1305 cm^−1^ is attributed to maleate species, which is subsequently oxidized to CO_2_ and H_2_O. Based on the *in situ* DRIFTS spectra results and the above analysis, it can be seen that benzyl alcohol, benzoate, maleate, CO_2_, and H_2_O were produced during the toluene oxidation over the MnO_*x*_/IM catalyst. Notably, the DRIFTS peaks of the 0.9/MnO_*x*_/IM and 1.5/MnO_*x*_/IM samples were weak at the initial reaction time. As the reaction proceeded, the peak intensities of the intermediates were significantly higher, especially benzoate, suggesting that the benzoate oxidation is the rate-determining step over the MnO_*x*_/IM catalyst. However, all reaction intermediates showed only weak accumulation on the 1.2/MnO_*x*_/IM catalyst, indicating that toluene oxidation proceeded rapidly.

**Fig. 7:**
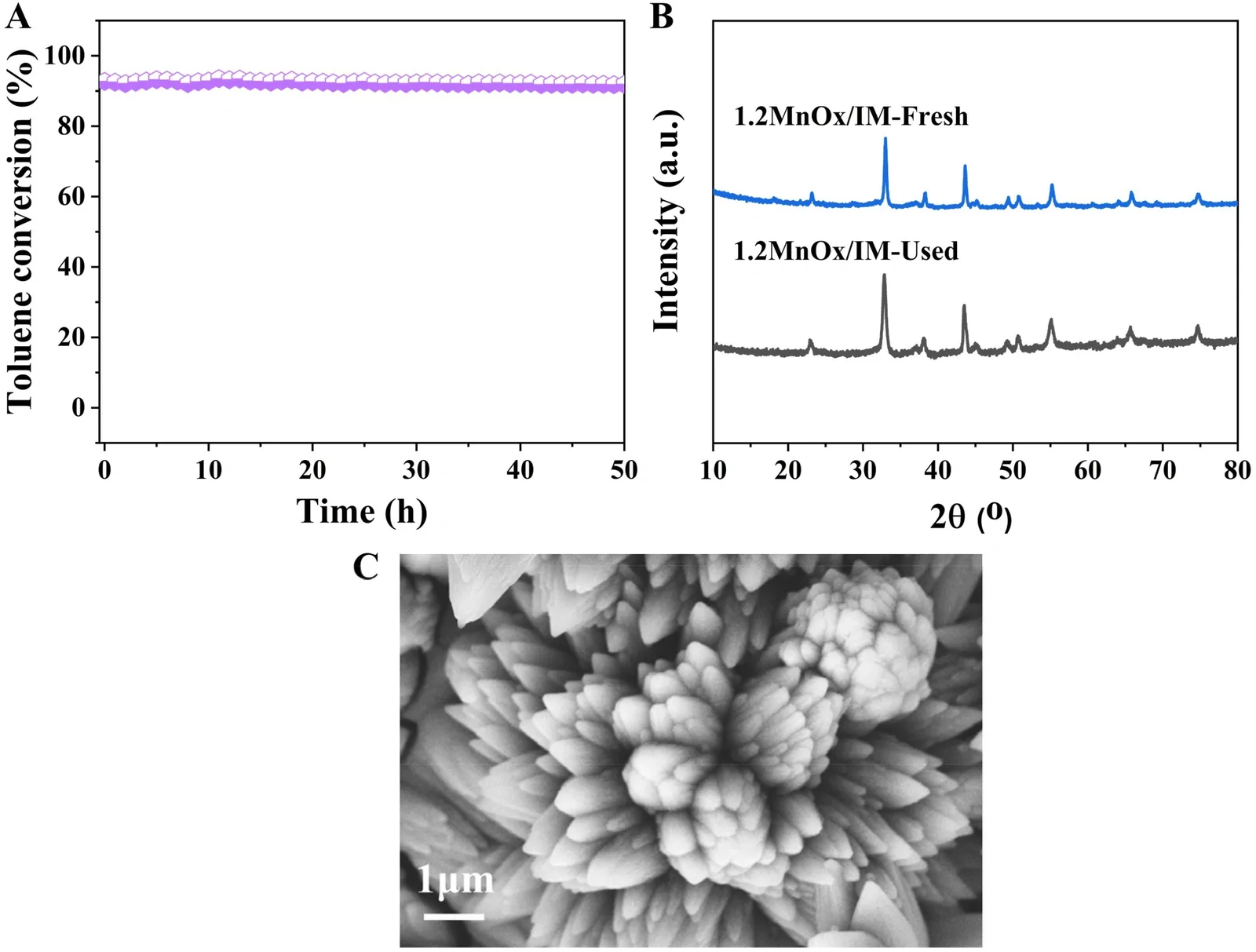
The stability result of the 1.2MnO_*x*_/IM catalyst at 310 °C (A). XRD patterns of fresh and used 1.2MnO_*x*_/IM catalyst (B). SEM image of the used 1.2MnO_*x*_/IM catalyst after reaction (C).

**Fig. 8:**
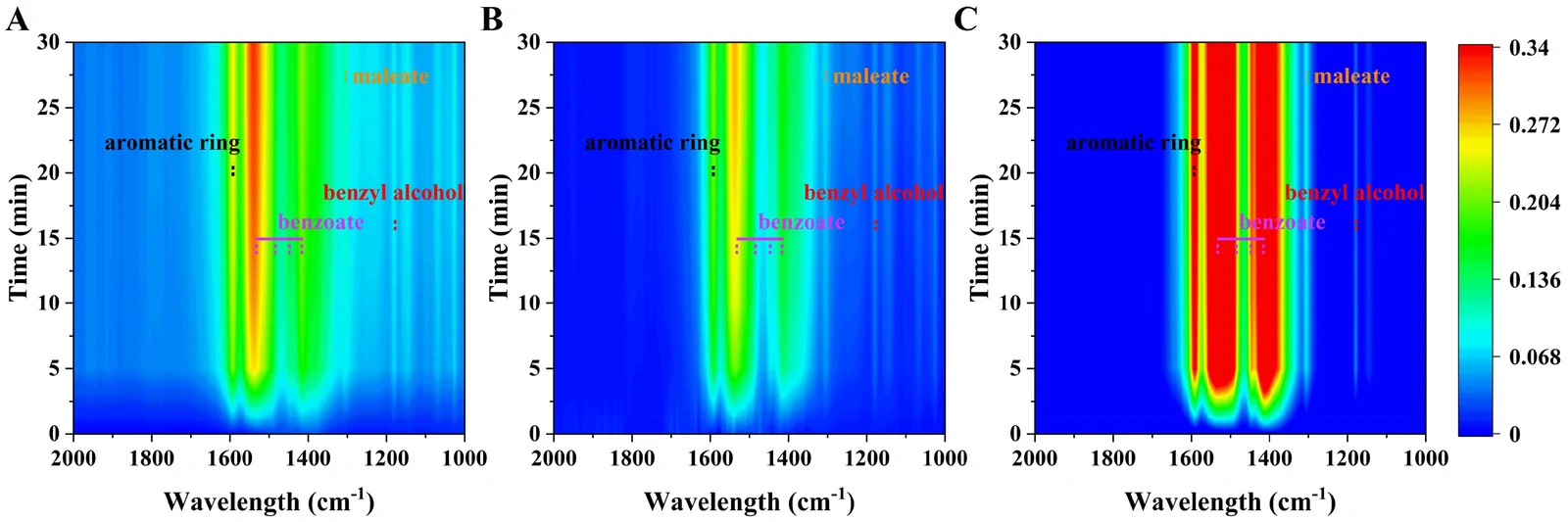
*In situ* DRIFTS spectra of 0.9MnO_*x*_/IM (A), 1.2MnO_*x*_/IM (B), 1.5MnO_*x*_/IM (C).

### 3.5 DFT calculations

The 1.2MnO_*x*_/IM catalyst showed predominant exposure of the Mn_2_O_3_ (111) crystal plane by XRD and HRTEM; as indicated by XPS and Raman, a high concentration of Mn^3+^ species and oxygen vacancies, thus exhibiting excellent catalytic activity for toluene oxidation. However, from the catalytic activity results in Fig. 6A, it can be observed that the 1.2MnO_*x*_/IM catalyst with an exposed Mn_2_O_3_ (111) plane shows only slightly better catalytic performance than the 0.9MnO_*x*_/IM catalyst with exposed Mn_2_O_3_ (100) planes. Thus, it is essential to demonstrate why the Mn_2_O_3_ (111) plane exhibits good catalytic activity for toluene oxidation.

To address this problem, the adsorption energy of two Mn_2_O_3_ crystal planes was calculated by the DFT method and shown in Fig. 9. Toluene and O_2_ bind more strongly to Mn_2_O_3_ (111) (*E*_ads_ = −1.24 and −0.76 eV) than to Mn_2_O_3_ (100) (−0.89 and −0.21 eV), suggesting the stronger adsorption of toluene and O_2_ on the Mn_2_O_3_ (111) plane. Introducing Ov markedly strengthens O_2_ binding on both facets, with −3.51 eV on (111) and −3.38 eV on (100), indicating that the (111) facet, with its stronger adsorption and activation capability, is the more active surface. Moreover, Mn_2_O_3_ (111) exhibits the lowest CO_2_ adsorption energy, facilitating its desorption and thereby releasing more active sites, improving both catalytic stability and subsequent cycling performance. These represent intrinsic electronic properties of the (111) facet of Mn_2_O_3_.

**Fig. 9:**
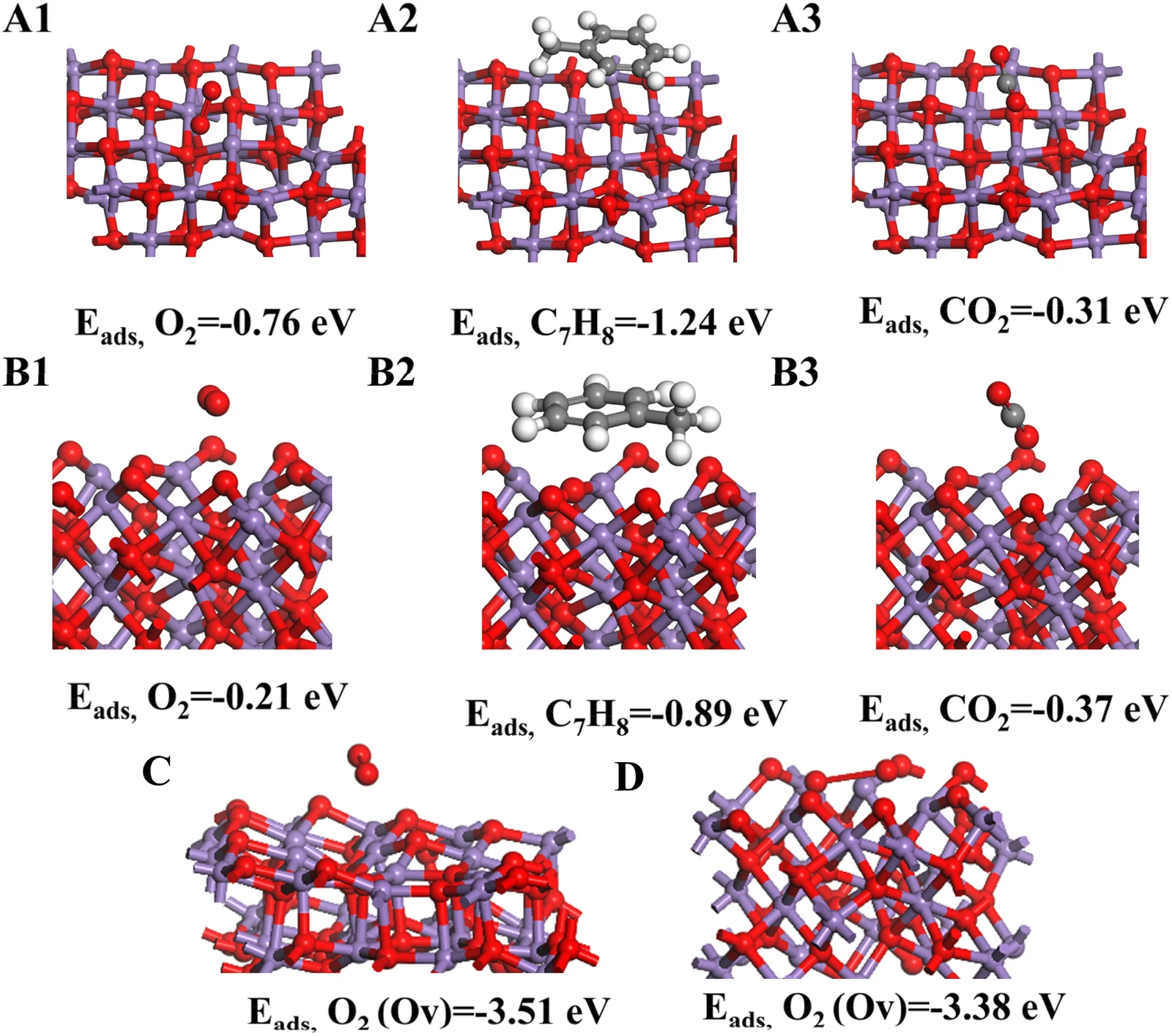
Adsorption configuration and adsorption energies of O_2_, toluene and CO_2_ on the Mn_2_O_3_ (111) (A) and Mn_2_O_3_ (100) (B) surfaces, and adsorption configuration and adsorption energies of O_2_ at oxygen-vacancy (O_v_) sites on Mn_2_O_3_ (111) (C) and Mn_2_O_3_ (100) (D).

## 4. Conclusions

In conclusion, a series of MnO_*x*_ catalysts was prepared to expose different crystal planes by varying the Mn(NO_3_)_2_ concentration during hydrothermal preparation. The superior activity and stability of 1.2MnO_*x*_/IM stem from the synergistic effect induced by the exposed Mn_2_O_3_ (111), *i.e.*, enriched oxygen vacancies, and good low-temperature redox ability. The Mn_2_O_3_ (111) crystal plane exhibited high toluene/O_2_ adsorption energy and low carbon dioxide adsorption energy, as demonstrated by DFT calculations, which are beneficial for toluene adsorption and carbon dioxide release, thereby enhancing the catalytic performance of toluene oxidation. The work opens the exploration of the role of the crystal plane in monolithic catalysts and provides a basis for the industrial application of metal-mesh-based monolithic catalysts.

## Author contributions

Kewang Huang: methodology, investigation, writing – review & editing. Jianshen Wang: methodology, formal analysis, writing – review & editing. Gangguo Zhao: software, investigation, data curation. Xiaojing Gui: methodology, investigation. Dan Liu: conceptualization, writing – review & editing, supervision. Jianzhou Gui: investigation, resources, supervision, project administration, funding acquisition.

## Conflicts of interest

The authors declare that they have no known competing financial interests or personal relationships that could have appeared to influence the work reported in this paper.

## Supplementary Material

RA-OLF-D6RA07826E-s001

## Data Availability

The authors confirm that the data supporting the study findings are available within the manuscript. Raw data that support these findings are available from the corresponding author upon request. Supplementary information (SI): detailed calculation methods for evaluating catalytic performance, DFT computational details, N_2_ adsorption–desorption results, high-resolution transmission electron microscopy (HRTEM) images, X-ray photoelectron spectroscopy (XPS) spectra of fresh and used catalysts, and supplementary tables summarizing structural properties and surface chemical states. See DOI: https://doi.org/10.1039/d6ra07826e.
