# Supplementary material for: Insights into the effect of exposed Mn2O3 crystal planes on toluene oxidation over MnOx/IM monolithic catalysts
Source: RSC Adv. 2026 Nov 2. Online ahead of print. doi: 10.1039/d6ra07826e (PMC13726628; doi:10.1039/d6ra07826e)
Supplement: Supplementary file 1 [file RA-OLF-D6RA07826E-s001.pdf]

Supplementary Information (SI) for RSC Advances.
This journal is © The Royal Society of Chemistry

Supplementary Material

Insight into the effect of exposed Mn_2O_3 crystal planes on toluene oxidation over MnOx/IM monolithic catalysts

Kewang Huang[a,b], *Jianshen Wang*[c], *Gangguo Zhao*[a], *Xiaojing Gui*[b], *Dan Liu*[a*], *JianZhou Gui*[a,d,∗]

[a] State Key Laboratory of Separation Membranes and Membrane Processes, Tianjin Key Laboratory of Green Chemical Technology and Process Engineering, and School

[b] Ningxia Coal Industry Co. Ltd., CHN ENERGY, Yinchuan 750011, P. R. China

[c] Shandong Haihua Co., Ltd., Weifang, Shandong 262737, China

[d] School of Chemical Engineering and Technology, Tiangong University, Tianjin 300387, China

∗ Corresponding authors.

E-mail address: liudan@tiangong.edu.cn; guijianzhou@tiangong.edu.cn.

S1 The calculated method of toluene conversion, CO_2 yield, active energy, and turnover frequency

The toluene conversion (X) and CO_2 yield (Y) were calculated using equations (1) and (2);

$$X = \frac{I_{in} - I_{out}}{I_{in}} \times 100\% \qquad (1)$$

where I_{in} and I_{out} are toluene signal intensity before and after reaction, respectively.

$$Y = \frac{I_{CO_2}}{I_{OC}} \times 100\% \qquad (2)$$

where I_{CO_2} is the CO_2 signal intensity under a certain reaction temperature, I_{OC} is the CO_2 signal intensity after toluene was completely oxidized.

The reaction rate of toluene oxidation was calculated by the following equation:

$$r = \frac{Q \times X}{m} \qquad (3)$$

Where r denotes the reaction rate ($mol \cdot g^{-1} \cdot s^{-1}$), Q is the molar flow of toluene ($mol \cdot s^{-1}$), m is the mass of Mn_2O_3 (g).

The activation energy was obtained by the Arrhenius equation (4):

$$\ln r = \ln A - \frac{E_a}{RT} \qquad (4)$$

Where Ea is the activation energy (kJ·mol-1), T is the reaction temperature (K), A is a pre-exponential factor, and R is the molar gas constant (8.314 $J \cdot mol^{-1} \cdot K$).

The turnover frequency (TOF) was obtained via the equation;

$$TOF = \frac{Q \times X \times M}{m} \qquad (5)$$

Where M is the relative molecular mass of Mn_2O_3.

S2 Characterization

Inductively coupled plasma emission spectrometers (ICP-OES) were tested on a Prodigy spectrograph. X-ray diffraction (XRD) patterns were recorded on the Ultima IV diffractometer, scanning from 10 to 80° using Cu Kα radiation with a tube voltage of 40 kV and a current of 40 mA. Scanning electron microscopy (SEM, JEOL S-4800) and high-resolution transmission electron microscopy (HRTEM, JEM-F200) images were recorded. Raman spectra were obtained using an XploRA PLUS Laser Confocal Raman spectrometer under the laser excitation of 532 nm. X-ray photoelectron spectroscopy (XPS) was performed on a Thermo Fisher Nexsa instrument. Temperature-programmed reduction (TPR) was performed on a Micromeritics Auto Chem II adsorption analyzer, equipped with a thermal conductivity detector. The detector signal was recorded in a 10% H_2/Ar stream at a flow rate of 30 mL/min, up to 700°C with a ramp rate of 10°C/min.

O_2 Temperature-programmed desorption (O_2-TPD) was carried out using *in-situ* mass spectrometry. For the test procedure, 100 mg of catalyst was placed in a quartz tube. First, the catalyst was pretreated under a flow of N_2 at 300℃ for 1 h to remove impurity gases, then cooled to below 50℃. A 21 vol% O_2/N_2 gas flow was introduced into the system for 1 h to ensure the catalyst reached O_2 saturation. Then, the gas was switched to N_2, and once the O_2 signal (m/z=32) had stabilized, the temperature was raised, and the O_2 signal was recorded.

In-situ DRIFTS was performed on a Nicolet iS50; the catalyst was loaded into an *in-situ* cell. It was first purged with N_2 at 300℃ for 1 hour, then cooled to room temperature to collect the background. After that, the reaction gas (1000 ppm toluene in a mixed gas of 21%

O_2 and 79% N_2) was introduced into the system. The spectra were collected at different temperatures within the range of 1000-2000 cm^{-1}. Especially, Spectra were collected by varying the reaction time at 310℃.

The adsorption energies of toluene, O_2, and CO_2 were calculated by DFT using the DMol3 module in Materials Studio. To elucidate the origin of the facet-dependent activity, spin-polarized DFT calculations were performed with the GGA-PBE functional and a DNP basis set, using a (2×2) supercell of the oxygen-terminated (O-terminated) Mn_2O_3(111) and (100) surfaces with four atomic layers, an adsorbate coverage of 0.25 ML, and explicit oxygen vacancies (Ov). To avoid interlayer interactions, the vacuum space along the Z direction was set to 15 Å.

$$E_{ads} = E_{cluster+species} - E_{cluster} - E_{species}$$

Where E_{ads} was the adsorption energy, $E_{cluster+species}$ and $E_{cluster}$ were the energies of the catalyst cluster with and without adsorbed species, respectively, and $E_{species}$ was the energy of toluene or O_2 adsorbed.

S3 The results of N_2 adsorption-desorption isotherms

In the N_2 adsorption-desorption isotherm (Fig. S1A), all samples present an IV-type isotherm with a hysteresis loop, indicative of the presence of mesopores. The pore size distribution in Fig. S1B can be observed to be larger than the most probable aperture of the 1.2MnOx/IM catalyst with an exposed Mn_2O_3 (111) surface, which is beneficial for the diffusion of reactant molecules. The specific surface area of IM and other MnOx catalysts, calculated by the BET method, is 0.4, 2, 2, and 3 m^2/g, respectively, suggesting that the surface area of the MnOx-supported catalysts is much larger than that of IM, indicating that more active sites are exposed on the MnOx catalysts.

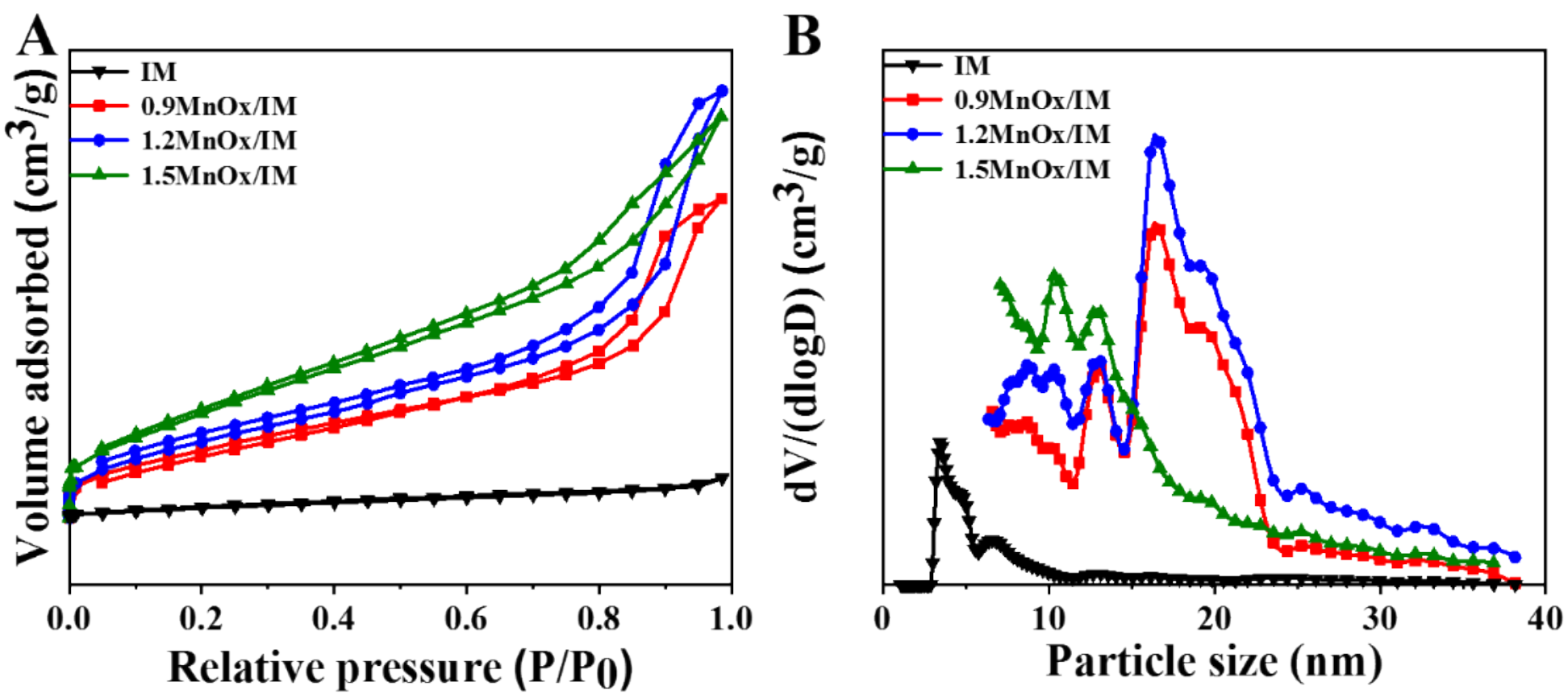

Fig. S1. N_2 adsorption/desorption isotherm (A) and pore size distribution (B) of all catalysts.

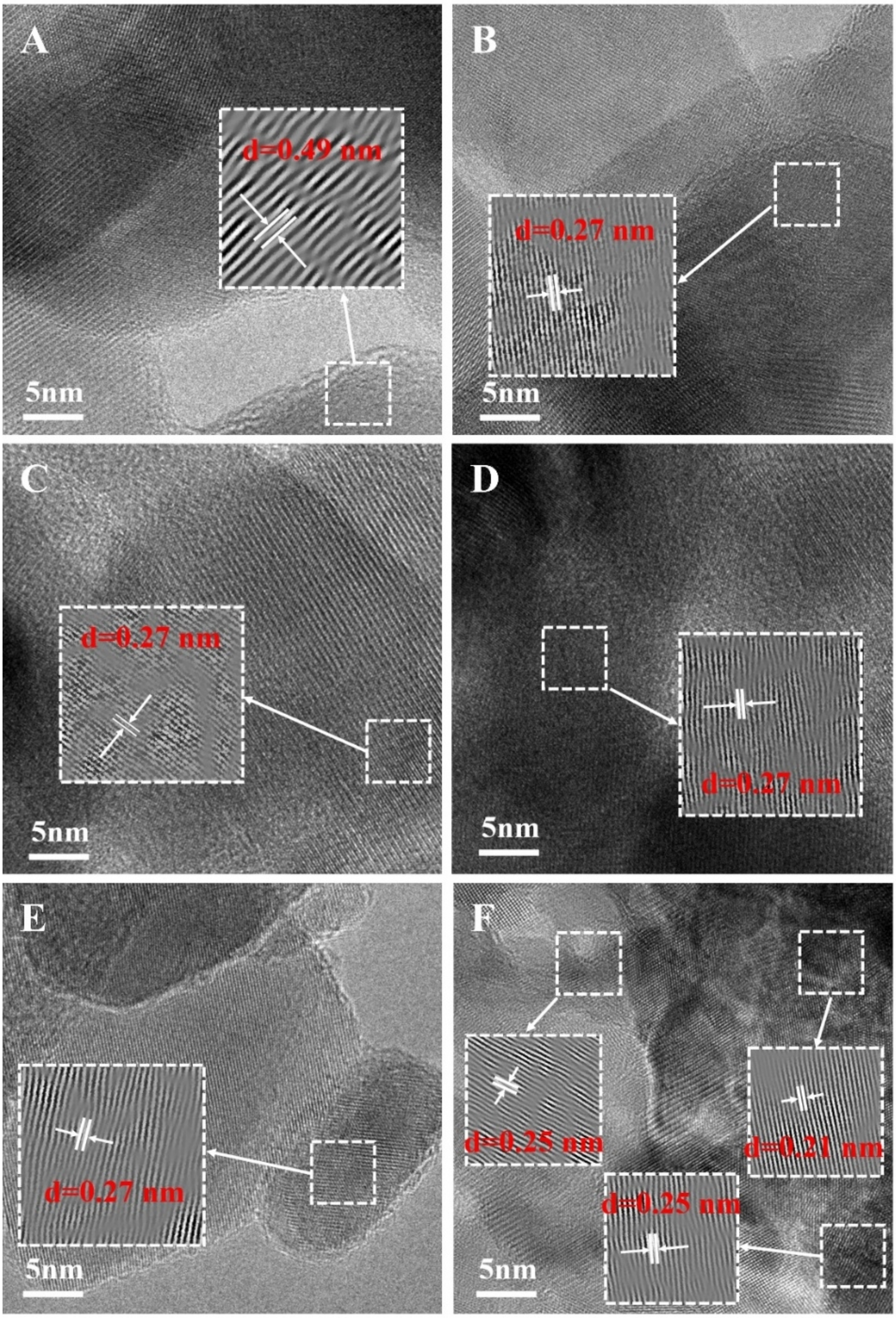

Fig. S2 HRTEM images of the catalysts 0.9MnOx/IM (A-B), 1.2MnOx/IM (C-E), 1.5MnOx/IM (F)

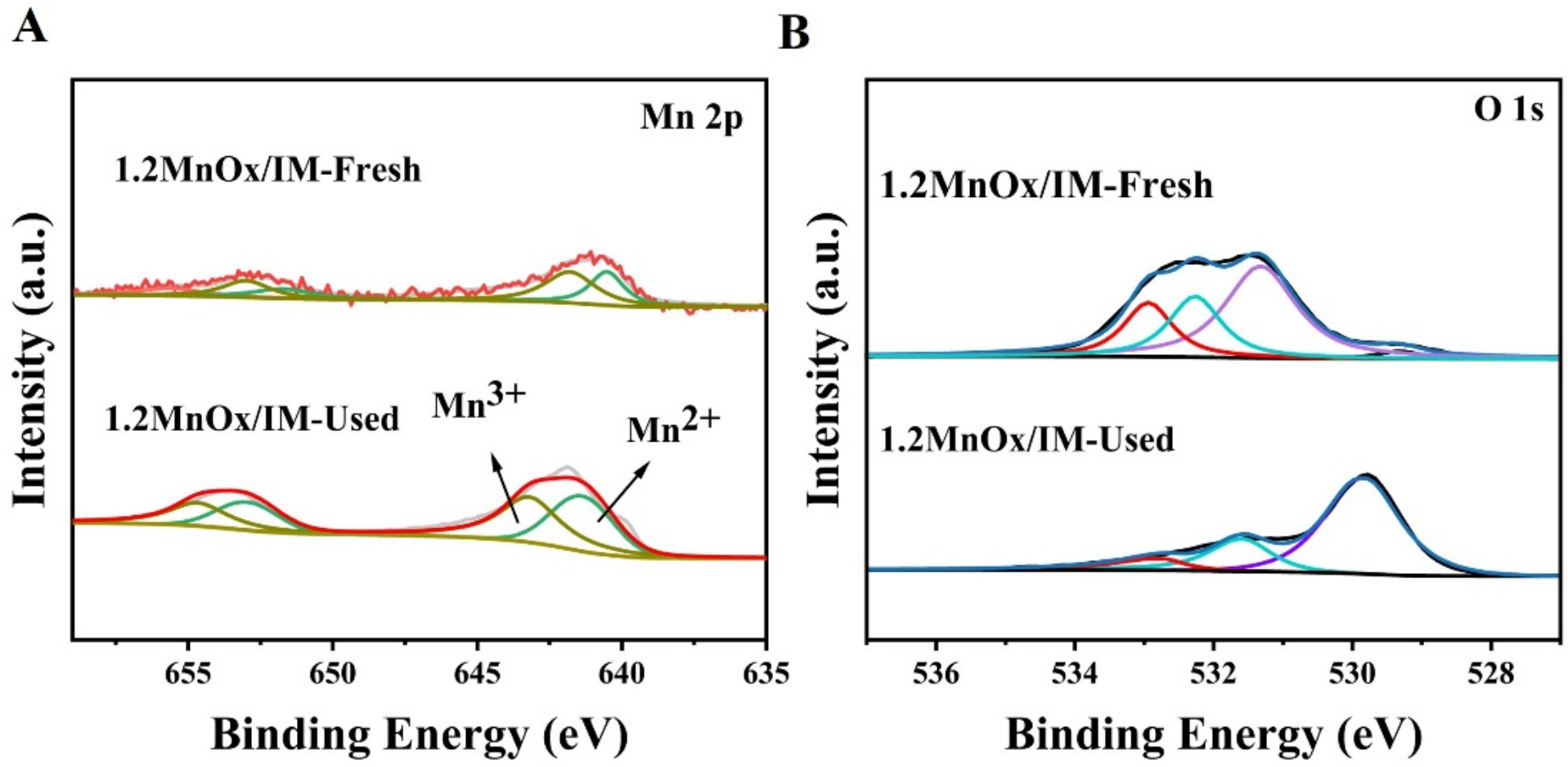

Fig. S3 Mn 2p (A) and O 1s (B) XPS spectra of the fresh and recycled 1.2MnOx/IM catalyst

Table S1 Surface area and pore diameter of the catalysts

Catalysts	Surface area (m^2/g) [a]	Pore diameter (nm) [b]
IM	0.4	3.1
0.9MnOx/IM	2	3.1
1.2MnOx/IM	2	3.8
1.5MnOx/IM	3	3.4

Notes: [a] Surface area was obtained by the BET method; [b] Pore diameter was calculated by the BJH method.

Table S2. Surface chemical states of the MnOx/IM catalysts.

Catalyst	Ov/O[a]	Mn^{3+}/Mn^{2+}
1.2MnOx/IM-Fresh	0.25	1.10
1.2MnOx/IM-Used	0.23	1.09

[a] O represents the total area of all oxygen species in O 1*s* XPS spectra of a certain catalyst.
